# Relationship between subjective socioeconomic status and sense of gain of health-care reform and the mediating role of self-rated health: a cross-sectional study in China

**DOI:** 10.1186/s12889-022-13106-y

**Published:** 2022-04-19

**Authors:** SU Yuan, LI Yueping

**Affiliations:** 1 School of Public Health, Fujian Medical University, Fuzhou, 350122 China; 2 School of Arts and Sciences, Fujian Medical University, Fuzhou, 350122 China

**Keywords:** Sense of gain of health-care reform, Subjective socioeconomic status, Self-rated health, Mediation effect

## Abstract

**Background:**

The sense of gain has gradually become the main evaluation index for the effectiveness of China’s deepening reform and is affected by many factors. However, there is no relevant research on the sense of gain of health-care reform (SGHR) and its influencing factors. The purpose of this study was to explore the influence of subjective socioeconomic status (SSS) on SGHR and the mediating role of self-rated health (SRH) between them.

**Methods:**

Data (25,149 samples total) from China Family Panel Studies (CFPS) in 2018 were included in the analysis. A nonparametric test was used to explore the differences in demographic characteristics of SGHR, and a correlation analysis and mediating effect model were used to explore the influence of SSS on SGHR and the mediating effect of SRH.

**Results:**

Demographic characteristics such as age, urban and rural areas, educational background, marriage and choice of medical treatment had significant differences in the distribution of perceived acquisition of medical reform. SSS, SRH and SGHR are statistically positively correlated with each other. SSS has a positive statistical correlation with SGHR, and may have an indirect effect through SRH.

**Conclusions:**

SSS is an important predictor of SGHR, and SRH may play a partially mediating role in SGHR.

## Background

“Difficult and Expensive Medical Treatment” is a serious health problem that has always existed in China and other countries. Since China launched the new health-care reform in 2009, a series of favourable policies have been introduced, and staged progress has been made including but not limited to the following aspects: hierarchical diagnosis and treatment, modern hospital management, universal medical insurance, drug supply guarantees and comprehensive supervision [1–4]. Important health indicators in China beyond the average level of high-income countries and objective indicators such as maternal mortality, infant survival rate and average life expectancy have shown that China’s health service capacity and health of the public have been improved [5]. Evidence from existing research suggests that China’s new healthcare reform offers valuable lessons for the world, especially reflected in the coverage and service quality of primary health care [6–8]. Although the Chinese government has continuously increased its investment in medical and health-care and various objective indicators have reflected the results of this comprehensive measure, what is the actual benefit to the public? Classical indicators such as satisfaction, well-being and subjective quality of life are typically used to measure the public’s subjective evaluation of the effectiveness of health services, but some experts believe that such indicators are too vague and often difficult to base themselves on objective actual needs, which is not in line with the current reform background in China [9–12]. Therefore, a suitable evaluation index is needed to explain how people feel about the dividend based on the results of health-care reform.

‘Sense of gain’, a social psychology concept to evaluate the effectiveness of a specific reform policy, has gradually become a research hotspot in the context of China’s comprehensive deepening of reform. This concept was used to evaluate the implementation effect of the reform from the subjective view of the public while emphasizing the objective material gain of the public, that is, the possession of public policy interests. Compared with classical concepts such as satisfaction and subjective well-being, sense of gain was of more practical significance in the current reform background of China [9–11, 13]. Moreover, some experts suggest that sense of gain had a positive prediction effect on happiness and satisfaction [14, 15]. The public’s sense of gain comes from the effective solution of existing social contradictions, which is usually reflected in the implementation of major livelihood infrastructure projects such as health-care, education, housing and the public environment, as well as the realization of social rights such as fairness and justice. However, current research on the sense of gain has not been specific to a certain field and primarily regards the overall sense of gain of social reform. Therefore, exploring the factors that may affect the public’s sense of gain in medical reform and their role can provide another perspective for evaluating the effectiveness of health-care reform implementation.

Socioeconomic status affects people’s behaviour patterns, psychological state, knowledge, resource acquisition ability and people’s sense of gain, which is usually measured from two aspects: objective socioeconomic status (SES) and subjective socioeconomic status (SSS). Relevant studies have shown that SES has a positive effect on SGHR. For example, Xiang’s empirical research shows that years of education, occupational status and income all have a significant positive impact on the sense of gain, and occupational status also has a positive impact on the sense of gain of intergenerational mobility [16]. Using longitudinal data, Lu found that with the improvement of personal living standards and subjective socioeconomic status, the sense of gain also increases [17]. Based on social comparison theory and expectation theory, Wang and Run et al. found that when people compare their SSS with their past or peers, the higher their evaluation of their current socioeconomic status, the stronger their sense of gain [18]. Sun’s research confirmed that the higher the socioeconomic status, the higher the urban residents’ sense of gain, and the predictive effect of SSS on the sense of gain was stronger than that of SES [19]. In addition, based on the positive prediction effect of the sense of gain on subjective well-being or satisfaction, relevant studies provide indirect evidence for this finding: people with low socioeconomic status also have low happiness and satisfaction and are prone to depression, anxiety, despair and even negative psychological states and behaviours such as self-harm and suicide [20–25]. Conversely, people with higher SES have more sound social functions, higher positive emotions and thus more positive cognitive evaluation of society [26]. As the result of health-care reform is an important source of the public’s sense of gain, it can be speculated that SES also positively affects the sense of gain of health-care reform (SGHR).

SGHR are affected not only by socioeconomic status but also by other factors such as an individual’s health status. Empirical studies show that self-rated health significantly (SRH) affects residents’ SGHR, and residents with lower health levels have a significantly lower SGHR than residents with higher health levels [27]. Relevant studies provide indirect evidence that SRH level is closely related to satisfaction and subjective well-being [28]. For example, Nader’s cross-sectional survey of residents in western Iran showed that residents with higher SRH have higher life satisfaction and both physical and mental health [29]. Data from the Canadian Community Health Service Survey showed that with the improvement of residents’ SRH, life satisfaction improved correspondingly, and self-rated mental health had a greater positive predictive power on life satisfaction [30]. Data from a survey on the oral health of children and adolescents in Lithuania showed that adolescents with a poor self-perception of oral health were more likely to report lower subjective well-being [31].

Although SRH has an impact on sense of gain, it may be also affected by SES. People with higher SES have a higher level of health, which is reflected in physical, psychological and social adaptation. Compared with SES, SSS is more effective in predicting the health level [32, 33], which can better reflect individuals’ sense of belonging to a certain social class, future prospects, social phenomena and job opportunities, as well as their attitudes and behaviours toward themselves and others. This trend is reflected in the above and earlier studies [34–36]. Therefore, this study explores whether SRH has a potential effect on the relationship between SSS and SGHR. Although some studies explored the potential influencing way of residents’ sense of gain and socioeconomic status from the perspective of community identity [37], there are few research on the potential influencing factors and what role they play in a specific field such as health-care reform.

To provide more sufficient evidence for relevant studies on the influencing factors of SGHR, this study proposes the following two hypotheses:

- **Hypothesis 1**. SSS may have a positive association with SGHR.
- **Hypothesis 2**. SRH may play a mediating role between SSS and SGHR.

**Fig. 1:**
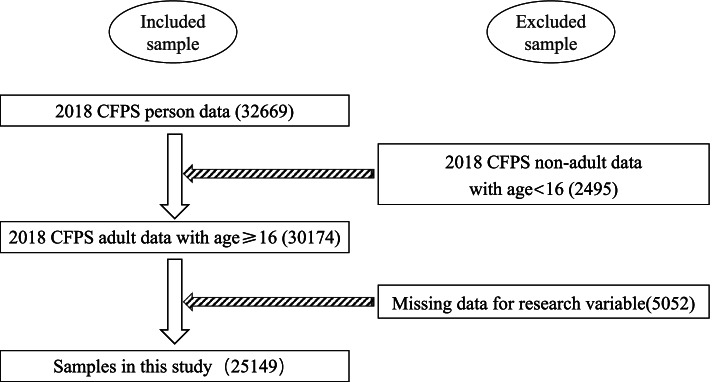
Hypothetical model of mediation effects

The hypothetical model relationships are shown in Fig. 1.

If a mediation effect is to be established, then it needs to meet the following requirements [38]: (1) The SSS must influence SGHR, that is, the coefficient c is significant (path c). (2) The SSS must affect the SRH, that is, the coefficient a is significant (path a). (3) When SSS, SRH and SGHR are included in the model at the same time, the influence of SRH on the SGHR must be significant (path b), and the influence of the SSS on the SGHR must be less than Eq. 1 (path c’); that is, the significance level or coefficient (absolute value) of coefficient c’ decreases compared with coefficient c. When the significance level of coefficient c’ decreases or the coefficient (absolute value) decreases, the part of the mediation function of SRH is verified. When the significance level of coefficient c’ disappears completely, the complete mediating effect of the mediator variable is verified. That is, when SRH is controlled, the SSS has no effect on SGHR.

## Methods

### Data

**Fig. 2:**
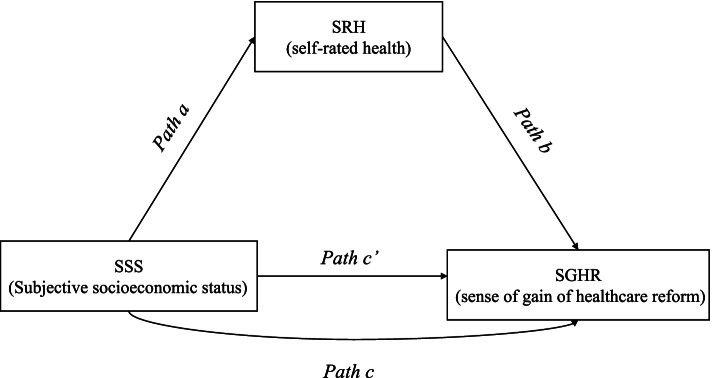
Processing of sample screening

The data used in this study were from the China Family Panel Studies (CFPS), which is a national and comprehensive social tracking survey project that reflects changes in Chinese society, economy, population, education and health [39]. The survey’s baseline sample covers 25 provinces/municipalities/autonomous regions, representing 95% of China’s population. The survey has been followed every 2 years since the baseline survey in 2010, with four wave tracking data thus far. In this study, individual data in the 2018 CFPS survey were used and updated in November 2019. After excluding non-adult data and missing data of variables concerned, including students, the final sample size was 25,149. SGHR, SRH, SSS and demographic characteristics were the main information in this study. Figure 2 shows the data for the data processing flow.

### Measures

#### Sense of gain of health-care reform (SGHR)

In this study, people’s subjective evaluation of the current status of China’s health-care reform in the questionnaire was taken as people’s SGHR. The corresponding question was, ‘How serious do you think our country’s medical problems are?’ The ratings ranged from 0 to 10, with higher scores indicating more serious medical problems. We reverse reset the score of this item: the higher the score, the less serious the health-care problems that are considered, and the higher the SGHR.

#### Subjective socioeconomic status (SSS)

According to the MacArthur scale of subjective social status, SSS is measured by two dimensions: evaluation of one’s own status in the community and evaluation of one’s own social status [40, 41]. The 2018 CFPS Adult Questionnaire included two questions: ‘Where is your income locally?’ and ‘What is your position in society?’ Both items were scored on a scale of 1 to 5; the higher the score, the higher the individual’s perceived income position or social status. Referenced to existing study, we combined the two, and scores were added to form scores of subjective socioeconomic status [42]. The higher the score, the higher the subjective socioeconomic status.

#### Health status

In this study, SRH was used as an indicator to evaluate individual health status. This indicator can even predict and measure long-term mortality risk, reflecting not only disease status but also health level in many aspects [41, 43, 44]. Respondents were asked, ‘How do you feel about your health?’ and were asked to choose one of five categories on a scale of one to five: extreme healthy, very healthy, relatively healthy, average or unhealthy. We also gave the result a reverse assignment: the higher the score, the better the self-rated health status.

#### Covariates

Other individual-level control variables include age, gender, marital status, education, residence and household status. According to Chinese age classification standards, we divided different age groups into youth, middle and old. Taking into account basic health, we also included indicators such as basic choice of care, trust in doctor, satisfaction with care and type of medical insurance. The descriptive analysis of the variables included in this paper is shown in Table 1.

**Table 1:** Descriptive analysis of the demographic characteristics of sample (*N* = 25,149)

Variable	N	%	Mean	SD
**Age**			48.24	15.71
Young (16-44)	10,130	40.28		
Middle (45-59)	8194	32.58		
Old (≥60)	6825	27.14		
**Gender**				
Male	12,593	50.07		
Female	12,556	49.93		
**Residence**				
Rural	12,449	49.70		
Urban	12,650	50.30		
**Household registration**				
Agricultural account	18,635	74.10		
Non-agricultural account	6514	25.90		
**Education**				
Primary school and below	10,864	43.20		
Junior middle school	7409	29.46		
High school	3749	14.91		
University/college and above	3127	12.43		
**Marital status**				
Never married	2090	8.31		
Married with spouse present	21,104	83.92		
Cohabitated	103	0.41		
Divorced	491	1.95		
Widowed	1361	5.41		
**Choice of medical institution**				
Clinic	5664	22.52		
Community health service station/Village clinic	3753	14.92		
Community health service centre// Township hospital	5350	21.27		
Special hospital	1430	5.69		
General hospital	8952	35.60		
**Type of medical insurance**				
Public medical insurance	630	2.51		
Urban employee basic medical insurance	4154	16.52		
Urban resident basic medical insurance	2234	8.88		
Supplementary medical insurance	164	0.65		
New rural cooperative medical insurance	17,967	71.44		
**Satisfaction with medical institutions**			3.32	0.97
**Trust in doctors**			6.73	2.38
**SSS**			6.04	1.86
**SRH**			1.94	1.21
**SGHR**			3.33	2.71

*Note*: *SSS* Subjective Socioeconomic Status, *SGHR* Sense of Gain of Health-Care Reform, *SRH* Self-Rate Heal, *SD* Standard Deviation

### Statistical analysis

SPSS 25.0 software was used for statistical analysis in this study: (1) Descriptive analysis was used to provide a simple report of various variables in this study. Numerical variables are shown in terms of the mean and standard deviation, and classified variables are shown in terms of proportion and quantity, as listed in Table 1. (2) The Harman single-factor test was used to judge whether there is common method bias among the major related questions in the questionnaire. If the explanatory power of the extracted first common factor is less than 40%, then there is no serious method bias among the questions. (3) Since the distribution of SGHRs is not normal (skewness = 0.560), this study uses the independent sample test of the nonparametric test to explore the differences in SGHRs of each classification variable, and pairwise comparisons are conducted among multiple classification variables. (4) Correlation analysis includes the three key variables in the mediation model and preliminarily explores the relationships among them. (5) Referring to the mediating effect model and test method [38, 45, 46], the bootstrap plug-in program PROCESS v3.0 by Andrew F. Hayes in SPSS is used to verify the mediating effect of self-rated health on subjective socioeconomic status and perceived gain of medical reform.

## Results

### Common method bias test

Three key variables (SSS, SRH and SGHR) were included in the test. In this study, unrotated exploratory factor analysis extracted two factors with characteristic roots greater than 1. The first common factor extracted in this study was SSS, which accounted for 37.78% of the total variance explanation. Therefore, it is believed that there is no serious methodological deviation among key variables [47].

### Analysis of differences in SGHR demographic characteristics

Nonparametric test results showed (Table 2) that the difference analysis of some categories had statistical significance, with different internal differences.

**Table 2:** Analysis of demographic differences in SGHR (Nonparametric Test)

Variable	Z/K^a^	*p-value*
**Age**	573.788^Z^	< 0.001
Young-Middle	-12.579^Z^	< 0.001
Young-Old	−23.762^Z^	< 0.001
Middle-Old	−11.167^Z^	< 0.001
**Gender**	5.210^K^	< 0.001
**Residence**	−16.692^K^	< 0.001
**Household register**	−17.374^K^	< 0.001
**Marital status**	61.015^Z^	< 0.001
Divorced- Never married	3.538^Z^	0.004
Divorced-Married with Spouse Present	4.105^Z^	< 0.001
Divorced-Cohabitated	0.638^Z^	1.000
Divorced-Widowed	−6.922^Z^	< 0.001
Never married- Married with Spouse Present	−0.456^Z^	1.000
Never married- Cohabitated	−1.068^Z^	1.000
Never married-Widowed	−5.381^Z^	< 0.001
Married with Spouse Present- Cohabitated	−1.198^Z^	1.000
Married with Spouse Present-Widowed	−6.328^Z^	< 0.001
Cohabitated-Widowed	−2.899^Z^	1.000
**Education level**	627.150^Z^	< 0.001
University/college and above-Primary school and below	20.144^Z^	< 0.001
University/college and above-Junior middle school	8.397^Z^	< 0.001
University/college and above-High school	2.778^Z^	0.033
High school-Primary school and below	18.031^Z^	< 0.001
High school-Junior middle school	5.684^Z^	< 0.001
Junior middle school -Primary school and below	15.106^Z^	< 0.001
**Choice of medical institution**	177.412^Z^	< 0.001
General hospital-Clinic	− 0.181^Z^	1.000
General hospital-Community health service station/Village clinic	−8.677^Z^	< 0.001
General hospital-Community health service centre// Township hospital	−9.041^Z^	< 0.001
General hospital-Special hospital	−3.299^Z^	< 0.001
Clinic-Community health service station/Village clinic	7.870^Z^	< 0.001
Clinic-Community health service centre// Township hospital	8.033^Z^	< 0.001
Clinic-Special hospital	3.279^Z^	0.010
Community health service station/Village clinic-Community health service centre// Township hospital	0.587^z^	1.000
Community health service station/Village clinic-Special hospital	8.404^Z^	< 0.001
Community health service centre// Township hospital - Special hospital	8.453^Z^	< 0.001
**Type of medical insurance**	366.139^Z^	< 0.001
Urban employee basic medical insurance-Public medical insurance	0.152^Z^	1.000
Urban employee basic medical insurance-Urban resident basic medical insurance	−3.688^Z^	0.002
Urban employee basic medical insurance-Supplementary medical insurance	−2.119^Z^	0.341
Urban employee basic medical insurance-New rural cooperative medical insurance	−17.156^Z^	< 0.001
Public medical insurance-Urban resident basic medical insurance	−2.001^Z^	0.454
Public medical insurance-Supplementary medical insurance	−1.850^Z^	0.643
Public medical insurance-New rural cooperative medical insurance	−7.126^Z^	< 0.001
Urban resident basic medical insurance-Supplementary medical insurance	−0.889^Z^	1.000
Urban resident basic medical insurance-New rural cooperative medical insurance	−8.852^Z^	< 0.001
Supplementary medical insurance-New rural cooperative medical insurance	−1.615^Z^	1.000

*Note*: ^a^indicates that the K value is Mann-Whitney test statistics, used for dichotomous variables including gender, residence and household registration; The Z value indicates Kruskal-Wallis test statistics for the remaining multiclassification variables in the table

This is embodied in the following aspects:

1. SGHR of women is higher than men’s.
2. The SGHR of the elderly is significantly higher than that of young people, and the older have the highest SGHR.
3. Rural residents have a higher SGHR than urban residents, and the difference in household register is almost the same as the difference in residence.
4. The results of marital status show that widowed people have the highest SGHR.
5. There is also a difference in SGHR reflected by different levels of education. In terms of the absolute value of the overall performance, the greater the educational gap, the greater the difference in the sense of gain. People with lower educational levels have higher SGHRs, while those with higher education levels have the lowest sense of gain.
6. In terms of the choice of medical institutions, those who choose general hospitals generally have a low SGHR, while those who choose primary medical institutions or specialized hospitals have a higher SGHR. In primary medical institutions, people who choose community health service centres or township health centres have higher SGHRs than those who choose clinics or health service stations/village clinics.
7. The SGHR of people participating in new rural cooperative medical insurance or supplementary medical insurance is higher than that of people participating in public medical insurance urban employee basic medical insurance or urban resident basic medical insurance.

### Correlation analysis

The results of Spearman correlation analysis showed (Table 3) that there was no correlation between satisfaction with medical institutions and SSS and SRH but there was a positive correlation with SGHR. The degree of trust in doctors was positively correlated with the other four variables, especially with regard to SSS. The correlation between the three variables in the mediation model was statistically significant. This met the basic conditions of the mediation effect analysis: SSS was positively correlated with SRH (*R* = 0.183, *P* < 0.01) and SGHR (*R* = 0.042, *P* < 0.01), and SRH was positively correlated with SGHR (*R* = 0.030, *P* < 0.01).

**Table 3:** Correlation analysis of main research variables

Variable	1	2	3	4	5
1.Satisfaction with medical institutions	1				
2.Trust in doctors	0.066**	1			
3.SSS	−0.002	0.157**	1		
4.SRH	0.014**	0.066**	0.183**	1	
5.SGHR	0.060**	0.069**	0.042**	0.030**	1

*Note*: *SSS* Subjective Socioeconomic Status, *SGHR* Sense of Gain of Health-Care Reform, *SRH* Self-Rate Health

*p < 0.05(2-tailed), **p < 0.01(2-tailed)

### Analysis of mediating effect

Using difference and correlation analyses (after controlling variables such as age, gender, marital status, education level, residence, household registration, choice of medical institution, satisfaction with medical institutions and trust in doctors), the hypothesis model was tested according to the mediation effect testing procedure proposed by Wen et al. [46]. The model size diagram is shown in Fig. 1. The results show that SSS was positively correlated with SGHR and SRH, respectively. At the same time, SRH also shows the same relationship with SGHR. The coefficient estimation results of the above relationship are reported in Table 4. The 95% confidence intervals of the bootstrap test results of the five path coefficients do not contain ‘0’, and the differences are statistically significant. Among them, the nonstandardized path coefficients of SSS and SRH to SGHR are 0.0425 and 0.0446, respectively. These values describe the direct effects of the two on the response to SGHR, corresponding to path c’ and path b, respectively. Simultaneously, SSS maybe have an indirect effect on SGHR through the intermediary variable SRH. In the total effect of SSS on SGHR (0.0475), the indirect effect of self-rated health is 0.0050 (path a * b); that is, the mediating variable of SRH mediates 10.53% of the effect, which is incomplete mediation or partial mediation.

**Table 4:** Test of mediation effects of SRH on relationship of SSS to SGHR: Bootstrap results

path/effect	Non-Standardized			
	Coef(β)	S.E.	LLCI	ULCI
C (Total effect)	0.0475	0.0092	0.0295	0.0655
a (SSS-SRH)	0.1111	0.0039	0.1034	0.1188
b (SRH-SGHR)	0.0446	0.0148	0.0156	0.0736
a*b (Indirect effect)	0.0050	0.0018	0.0016	0.0084
c’(Direct effect)	0.0425	0.0093	0.0242	0.0609

*Note*: *SSS* Subjective Socioeconomic Status, *SGHR* Sense of Gain of Health-Care Reform, *SRH* Self-Rate Health, *Coef(β)* Nonstandardized effect values of bootstrap results, *S.E* Standard Error, *LLCI* the lowest 95% confidence interval of bootstrap test results, *ULCI* the highest 95% confidence interval of bootstrap test results

*:The indirect effect of multiplying path a and b path

## Discussion

The new round of health-care reform in China has achieved remarkable results in all aspects, but it is not clear which factors affect people’s feelings about this effect and its possible impact paths. Using 2018 CFPS data, this study explored the differences in SGHRs under different demographic characteristics and proposed two hypotheses about the relationships among SSS, SRH and SGHR as well as their internal influencing paths based on relevant studies. The results of the mediating effect analysis support the hypothesis proposed in this study: SSS may have a positive association with SGHR., and SRH perhaps play a part in the mediating effect between the two.

In the difference analysis, this study found:

- Firstly, there are differences in perceived gain by gender and age. In particular, older people scored higher in the SGHR, especially in the elderly. A possible explanation is that elderly people over the age of 60 or older people experienced a long and complex process of change of medical and health system reform. Given this background, compared with those past, it is easy to feel that the present medical and health conditions, health-care and other aspects of the actual gain have improved. Teenagers, on the other hand, have not experienced hardships or hardships of their parents’ or grandparents’ time, and they have higher requirements for the health-care environment and stronger expectations of health returns. This is in line with Senik’s findings that compared with horizontal comparison with others, longitudinal comparison in the time dimension has a stronger impact on the personal perception of gain and loss [48]. This phenomenon can be extended to other areas of social reform and is not limited to the sense of access to medical reform [49–51].
- Secondly, rural people have a higher SGHR than urban people. On the one hand, urban people’s expectations of the medical level further improved, and the corresponding urban people’s demand for a certain medical level is much higher than the actual improvement of medical services. On the other hand, rural medical resources are relatively scarce, and more debt has existed for a long time. Since China’s new health-care reform, the government has increased investment in new rural cooperative medical care, and the primary-level medical and health conditions have changed to a great extent. This has improved rural people’s SGHR, especially doctor-patient communication and improvements to the medical institution environment [52, 53].
- Thirdly, medical reform benefits widowed people more, which is similar to Wang’s findings [37]. In general, widowed people receive more social assistance or welfare, including health assistance and especially economic benefits in health-care insurance.

Another interesting phenomenon is differences in educational background show that respondents with higher education have a lower sense of medical reform acquisition, which is contrary to the positive impact of SSS on SGHR in this study. Generally, a higher education level means higher socioeconomic status and thus a higher SGHR [54]. Such results may indicate that the evaluation or predictive efficacy of SES and SSS indicators is not entirely consistent [32–36]. At the same time, from another point of view, the young group with higher education is the main employment body in the city, and they bear higher medical insurance costs and medical expenses. Moreover, compared with the elderly, young people have higher expectations for the medical and health conditions around them and do not benefit much from medical reform. The uneven distribution of high-quality health resources aggravates the anxiety of this group, which reflects the realistic medical problem of ‘difficult and expensive medical treatment’.

In addition, in terms of the selection of daily medical institutions, the results show that compared with other medical institutions, those who often choose general hospitals have the lowest sense of obtaining medical reform, while those who often go to township health centres and community health service centres have the highest SGHR. Such results are consistent with the current status of China’s health-care reform. On the one hand, although China’s health-care reform has made remarkable achievements in all aspects, the phenomenon of ‘difficult and expensive medical treatment’ still exists, especially in general hospitals [4, 55, 56]. On the other hand, the coverage, content and quality of primary health services and public health services in China have been significantly improved with the support of health-care reform policies in recent years [1, 2, 4, 5, 8]. Thus, people benefit more from primary health institutions and thus have stronger SGHR.

Finally, we find that differences in SGHR among people with different medical insurance types. New rural cooperative medical insurance and supplementary medical insurance for the general health and economic burden of rural residents for a long time provides the help, at the same time, since the new healthcare reform is to expand the basic medical service coverage to a large extent, resolves the problem of people “ Difficult and Expensive Medical Treatment “, and to live in this part of the urban population, which is involved in urban employee basic medical insurance or public medical insurance or Urban resident basic medical insurance for urban residents of this part of the crowd, they are less likely to benefit from such benefits, which may explain why the SGHR is highest among those who participate in New rural cooperative medical insurance and supplementary medical insurance [57, 58]. The difference analysis results suggest that these demographic characteristics may have different degrees of influence on the public’s SGHR, so they should be considered as control variables to reduce the mixed impact of mediation analysis.

The results of the correlation analysis provide statistical support for us to explore the mechanism of action among SSS, SRH and SGHR; that is, the three are positively correlated with each other. Besides, the relationship between satisfaction with medical institutions and trust in doctors and the three key variables provides hints for the inclusion selection of control variables and may provide indirect evidence for the claim that the sense of gain can predict satisfaction.

Based on the above, the two hypotheses proposed in this study may be verified in the results of the mediation effect analysis.

One demonstration is that SGHR may association with SSS. The higher the SSS, the higher the SGHR, which is consistent with the general direction of existing studies [15, 17, 19]. However, as mentioned above, most of the existing studies focus on residents’ overall sense of gain from reform rather than the specific field of health reform. Hypothesis 1 is statistically reflected in the implementation of medical reform policies. Policy-makers need to attach importance to enhancing the public’s SES, especially SSS. Shortening the distance of SSS between people may be of great significance for enhancing the whole SGHR. These policies are not confined to health policy and still need to link to the macrosocial environment such as education, employment and other social security [16, 54, 59–61].

The other demonstration is that SRH probably has a positive effect on SGHR and may play a partial mediating role in the relationship between SSS and SGHR. As stated earlier, SRH is a comprehensive evaluation about people’s physiological health, psychological health and social adaptation level [41, 43, 44]. This is closely related to all kinds of satisfaction or happiness and is reflected in different ages and different health statuses of the crowd. At the same time, the higher SRH of people in the social evaluation is more active and positive [28–30, 62]. This is the key reason that the hypothesis was proposed in this study. In this mediating relationship, the results of this paper first support the classical relationship that SSS has a positive impact on SRH in previous studies. People with higher SSS have more social resources including health resources, which determines their higher health level and health literacy [34–36]. In addition, it is clear that people with higher SRH are more positive in their evaluation of the effectiveness of health-care reform. This suggests that SGHR is indeed closely related to the indicators of happiness and satisfaction [12, 14], but the specific relationship between SGHR and medical satisfaction needs to be supported by more rigorous investigation and evidence. Most important, SRH played a mediating effect of nearly 9%, indicating that SSS exerts an influence on SGHR through SRH in the process of influencing SGHR. In other words, people with strong SSS have higher SRH, which leads to stronger SGHR. Such a logical chain relationship suggests that politicians and scholars need to pay attention to people’s health status. Focusing only on social and economic status may easily fall into the trap of the ‘Easterlin Paradox’. In addition, the public’s SRH is a key factor that reflects whether the health-care reform policy is in place and whether the public gains benefits. This further suggests that SRH plays an important role in assessing and predicting people’s social adjustment.

### Strengths and limitations

The results of this study suggest that SSS should be considered an important influence when measuring perceived health reform outcomes, that is, SGHR. The other important result is that this study examined whether SRH mediates the relationship between SSS and SGHR. The above provides important clues for studying the influencing factors of SGHR or other public policies.

However, some limitations existed in this study. First, the measurement of SGHR itself is relatively weak, and it is difficult for us to understand its specific composition mechanism due to the limitation of the questionnaire content. We hope that there will be more data of this measurement method in other countries or regions with healthcare reform in the future to improve the representativeness and persuasiveness of this measurement method. At the same time, we also hope to provide some possible evidence and ideas for future in-depth research on the measurement method of SGHR. Second, the sample size of this study is large and may be representative to some extent, but the statistical significance may not reflect the practical policy significance. Further exploration is needed. For example, for people of different categories or in different states, age, educational background, marital status and other factors should be carefully considered and explored with appropriate statistical methods, which may reflect the practical significance of the results. Third, although the main demographic characteristics were taken into account in this study, important variables such as employment and number of family members were still missing due to the influence of personal questionnaire content. Meanwhile, the results suggest that there should be other important factors playing a role in the relationship between SSS and SGHR, and more evidence is needed for further investigation in the future. Finally, as a cross-sectional study, although the results of this paper suggest that SGHR may be positively or negatively affected by some demographic characteristics and relevant healthcare services, it is still difficult to confirm the causal relationship between SSS and SGHR, but at least it provides a direction for future research.

## Conclusion

In brief, this study explored the relationship between SSS and SGHR by using data from a large-scale cross-sectional survey across the country and tested the mediating effect of SRH between them. With the advancement of the health reform process, it is necessary to have an effect-based evaluation index based on the public’s standpoint to endorse it. The SSS and SRH of the public deserve the attention of health policy-makers and relevant scholars or should be included in the evaluation mechanism of the health-care reform policy effect.

## Data Availability

The data of the studies is publicly available and could be accessible via website: China Family Panel Studies(CFPS) (pku.edu.cn).

## Abbreviations

SES: Objective Socioeconomic Status
SSS: Subjective Socioeconomic Status
SGHR: Sense of Gain of Health-Care Reform
SRH: Self-Rate Health
CFPS: China Family Panel Studies
